# Synthesis and performance of solid proton conductor molybdovanadosilicic acid

**DOI:** 10.1039/c8ra02390e

**Published:** 2018-04-16

**Authors:** Zhirong Xie, Han Wu, Qingyin Wu, Limei Ai

**Affiliations:** Department of Chemistry, Zhejiang University Hangzhou 310027 P. R. China qywu@zju.edu.cn; School of Biomedical and Chemical Engineering, Liaoning Institute of Science and Technology Benxi 117004 Liaoning P. R. China

## Abstract

A molybdovanadosilicic acid H_5_SiMo_11_VO_40_·8H_2_O was synthesized and investigated in this work. The structure features and hydration degree of this acid were characterized by IR, UV, XRD and TG-DTA. Its proton conductivity was studied by electrochemical impedance spectroscopy (EIS). The EIS measurements demonstrated that H_5_SiMo_11_VO_40_·8H_2_O showed excellent proton conduction performance with proton conductivity reaching 5.70 × 10^−3^ S cm^−1^ at 26 °C and 70% relative humidity. So, it is a new solid high proton conductor. The conductivity enhances with the increase of temperature, and it exhibits Arrhenius behavior. The activation energy value for proton conduction is 21.4 kJ mol^−1^, suggesting that the proton transfer in this solid acid is dominated by Vehicle mechanism.

## Abstract 2

A novel Keggin-type proton conductor shows high proton conductivity, reaching 5.70 × 10^−3^ S cm^−1^ at room conditions.

## Graphical abstract

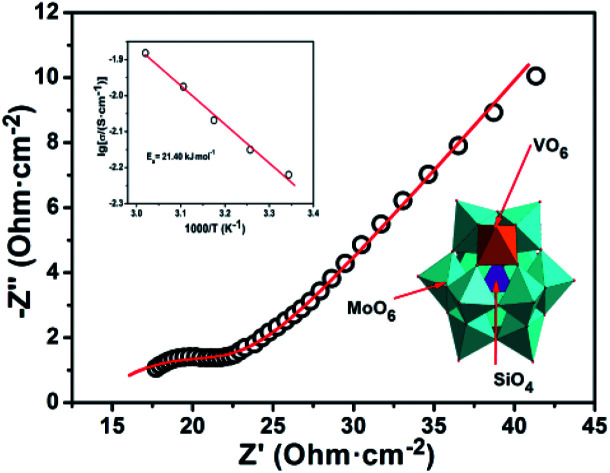

## 1. Introduction

Heteropoly acids (HPAs) are a unique class of nanosized polynuclear clusters composed of transition metals and oxygen atoms.^1,2^ During the past decades, HPAs have attracted huge interest owing to their huge potential in many fields, for instance, catalysts, biomedicine and material science.^3–7^ In particular, due to their high proton conductivity and proton transfer/storage abilities, HPAs have become one of the best candidate electrolyte materials for the development of fuel cells and supercapacitors.^8–12^ So, a further investigation of the role and function of these compounds in electrochemical devices will give insight into developing solid electrolytes based on HPAs.^13^ HPAs contain two kinds of protons: (1) dissociated hydrated protons connected to one heteropolyanion as a whole; (2) non-hydrated protons located on the bridge-oxygen or terminal-oxygen atoms of the polyanion.^14^

Keggin-type HPAs, which are an important branch of HPAs, occupy an unique place at the forefront of the HPAs field because of their many advantages, including chemical stability and convenience of synthesis.^15,16^ Its chemical formula can be expressed as [XM_12_O_40_]^*n*−^, with mainly X = P, As, Si and Ge, and M = W, Mo and V. What attracts us most is that the structure and proton conductive property of HPAs vary with the component elements of heteropolyanion changes. According to our recent researches,^17^ vanadium have great impact on the thermal stability and proton conductivity of heteropoly acids. Hence, in this work, based on Keggin-type HPAs, a mono-vanadium-substituted molybdovanadosilicic acid H_5_SiMo_11_VO_40_·8H_2_O is synthesized, and its structure, hydration and proton conductive properties have also been investigated.

## 2. Experimental section

### 2.1. Preparation of H_5_SiMo_11_VO_40_·8H_2_O

**Fig. 1:**
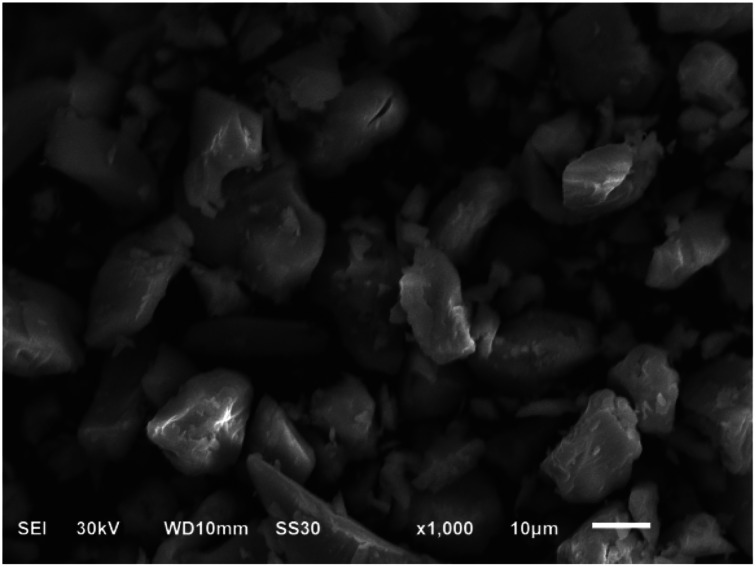
The SEM of H_5_SiMo_11_VO_40_·8H_2_O.

2.84 g of Na_2_SiO_3_·9H_2_O was dissolved in 100 mL deionized water, and added 26.62 g Na_2_MoO_4_·2H_2_O to the above solution. Then this mixed solution was heated to boil for 30 min. After that, 50 mL aqueous solution which contained 1.22 g NaVO_3_ was added to the mixture. The mixture was continuously heated at 90 °C for 2 h with the assistance of stirring under the condition of pH = 2.5 (adjusted by 1 : 1 H_2_SO_4_ solution). Lastly, the cooled solution was extracted with 30 mL ether in sulfuric acid environment. The powder H_5_SiMo_11_VO_40_·8H_2_O was obtained after the concentrated etherate solution was dried in vacuum. The color of the product is orange-red and the morphology of this solid is as shown in Fig. 1, which indicates that this heteropoly acid exhibits crystalline structure. The element contents of silicon, molybdenum and vanadium in H_5_SiMo_11_VO_40_·8H_2_O were collected by inductively coupled plasma mass spectrometer (ICP-MS). The content of water was figured out by thermogravimetry. Found: Si: 1.37%; Mo: 54.06%; V: 2.49% and H_2_O: 9.30%. Calculated for H_5_SiMo_11_VO_40_·8H_2_O: Si: 1.43%; Mo: 53.87%; V: 2.60% and H_2_O: 9.18%. The theoretical values are in good agreement with the experimental results.

### 2.2. Instructions and reagents

Element content was measured on a THERMO ELECTRON PQ3 inductively coupled plasma mass spectrometer (ICP-MS). The Fourier-transform infrared spectroscopy (FTIR) spectrum was performed on a NICOLET NEXUS470 FT/IR spectrometer using KBr as pellets, and the resolution is 4 cm^−1^. UV spectrum was got using SHIMADZU UV-2550 UV-Vis spectrophotometer.

Powder X-ray diffraction (XRD) pattern was recorded on a BRUKER D8 ADVANCE X-ray diffractometer in the range of 2*θ* = 3–40° at the rate of 0.02° s^−1^. The crystal data was collected using graphitemono-chromatic Mo–K radiation (0.71073 Å) at 293 K, and the data sets were corrected by empirical absorption correction using spherical harmonics, implemented in SCALE3 ABSPACK scaling algorithm. The thermal stability of the sample was carried out using simultaneous thermogravimetry (TG) and differential thermal analysis (DTA) technique, measurements were performed using a Shimadzu thermal analyzer in a nitrogen stream from room temperature to 600 °C, with the scanning rate of 10 °C min^−1^.

All chemicals were of analytical grade and used without further purification.

### 2.3. Measurement of proton conductivity

At ambient condition, the crushed sample was pressed at 25 MPa into a compacted pellet. The diameter of the obtained tablet is 10.00 mm and the thickness of it is 1.02 mm. The proton conductivity was measured using a cell: copper | sample | copper, in which copper slices were attached to the two sides of the tablet as electrodes. Complex impedance measurements were carried out on a VMP2 multichannel potentiostat electrochemical impedance analyzer over a frequency range from 9.99 × 10^4^ to 0.01 Hz.

## 3. Results and discussion

**Fig. 2:**
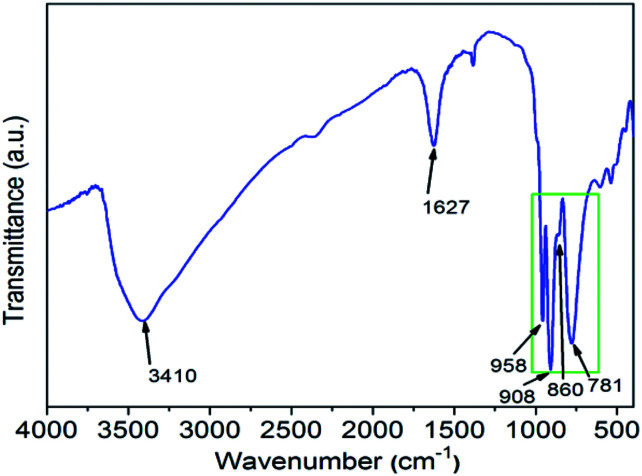
The IR spectrum of H_5_SiMo_11_VO_40_·8H_2_O.

IR is a useful method for investigating the structure information of heteropoly compounds. There are four kinds of oxygen atoms in [SiM_12_O_40_]^*n*−^. The SiO_4_ tetrahedron is located in the center of the twelve MO_6_ octahedra, which can be split into four groups of three edge-shared octahedra, M_3_O_13_. Furthermore, the M_3_O_13_ units are connected together by shared corners to each other. So, there are four kinds of oxygen atoms in [SiM_12_O_40_]^*n*−^: Si–O_*a*_, M–O_*b*_–M, M–O_*c*_–M and M–O_*d*_, where each separately represent inner oxygen, corner-sharing oxygen, edge-sharing oxygen and terminal oxygen. Fig. 2 presents that, at the range of 700–1000 cm^−1^, the IR spectrum of H_5_SiMo_11_VO_40_·8H_2_O exhibits four characteristic peaks. They are 958 cm^−1^, 908 cm^−1^, 860 cm^−1^ and 781 cm^−1^, which are assigned to *ν*_as_(M–O_*d*_), *ν*_as_(Si–O_*a*_), *ν*_as_(M–O_*b*_–M) and *ν*_as_(M–O_*c*_–M) vibration modes of heteropolyanion SiMo_11_VO_40_^5−^. For comparison, these four peaks of H_4_SiMo_12_O_40_ are 957 cm^−1^, 904 cm^−1^, 855 cm^−1^, 770 cm^−1^.^18^ The similarity of vibration bands demonstrates that the heteropoly acid H_5_SiMo_11_VO_40_ has the Keggin structure as its parent acid H_4_SiMo_12_O_40_ does.^19^ Obviously, all of these peaks have slightly shifted, which caused by the more negative charge carrier and the shift symmetry of molecular structure of H_4_SiMo_11_VO_40_·8H_2_O. Furthermore, there are two strong absorption peaks at 3410 cm^−1^ and 1627 cm^−1^ ascribed to the stretching vibration of O–H bond and the bending vibration of H–O–H bond.

**Fig. 3:**
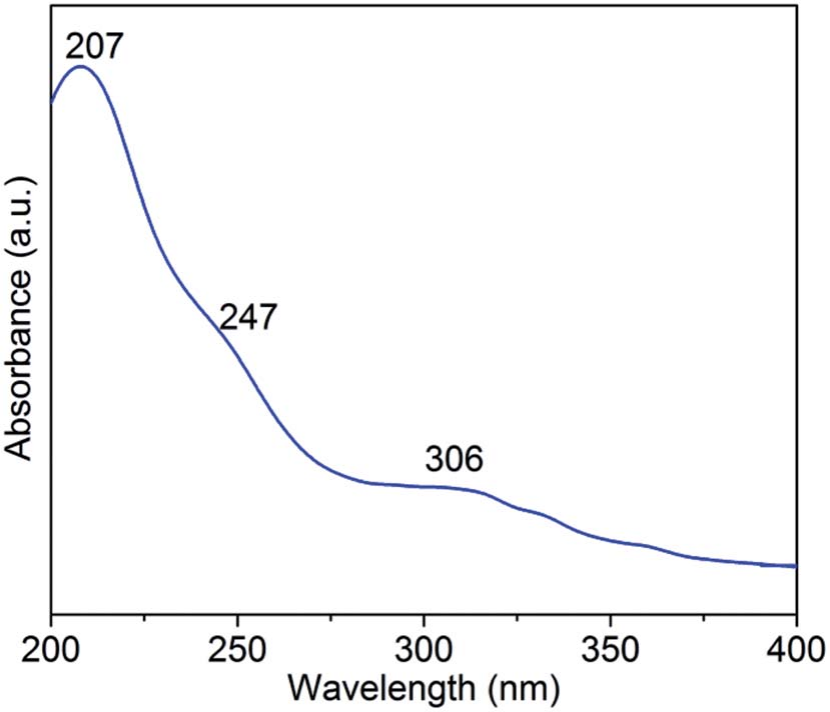
The UV spectrum of H_5_SiMo_11_VO_40_·8H_2_O.

The UV spectrum is very useful to distinguish the electronic properties of the metal ions. As shown in Fig. 3, there are three absorption bands identified in the UV spectrum of H_5_SiMo_11_VO_40_·8H_2_O. The intense peak at 207 nm belongs to the charge-transfer from terminal oxygen to metal atoms (O_*d*_ → M). The relatively weak bands at 247 nm and 306 nm are ascribed to the charge-transfer from bridge oxygen to metal atoms (O_*b*_/O_*c*_ → M). Inspired by the related literature,^20^ we could conclude that these absorption bands are the characteristic bands of Keggin-type heteropolyanion.

**Fig. 4:**
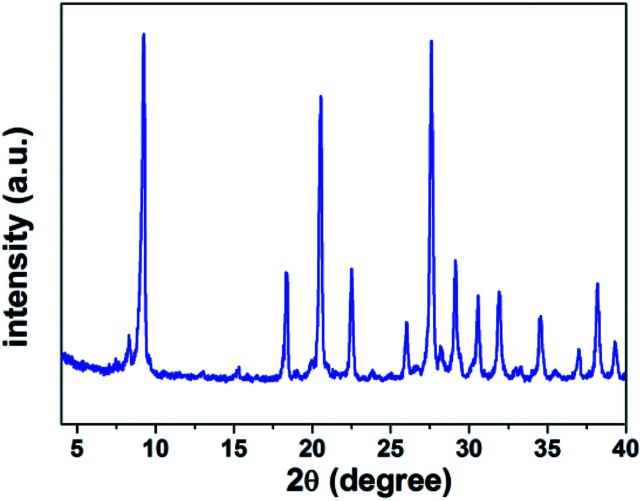
The XRD pattern of H_5_SiMo_11_VO_40_·8H_2_O.

**Fig. 5:**
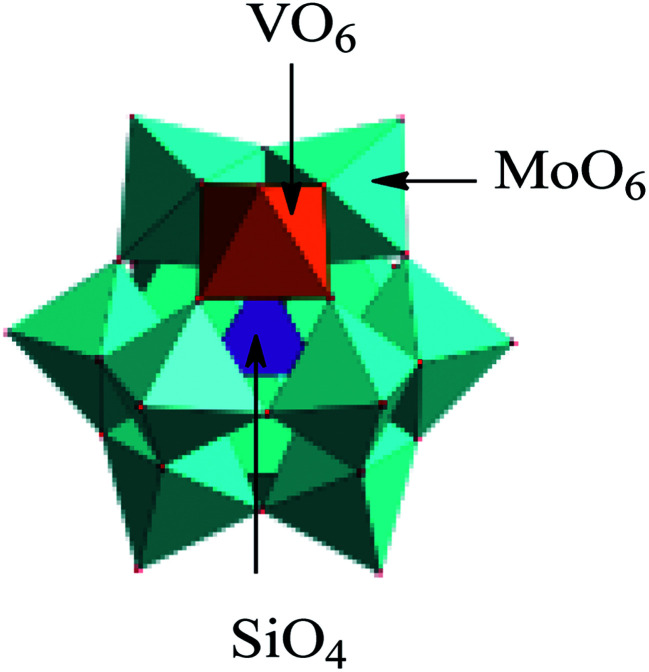
The structure of heteropolyanion SiMo_11_VO_40_^5−^. Color legend: VO_6_, orange octahedra; MoO_6_, green octahedra; SiO_4_, purple tetrahedra.

We have also got some information about crystal structure of H_5_SiMo_11_VO_40_·8H_2_O by powder X-ray diffraction (XRD). Fig. 4 is the powder X-ray diffraction pattern of H_5_SiMo_11_VO_40_·8H_2_O. From the data of XRD in Table 1, we get to know that the characteristic peaks of this HPA mainly exist at the range of 8–10°, 17–23°, 25–32° and 33–38°, and the most intensive peak is at about 9.25°. They are the characteristic peaks of HPAs with Keggin structure.^21^ Up to now, XRD, combined with UV and IR spectra, allows us to verify that the heteropoly acid H_5_SiMo_11_VO_40_ possesses the typical Keggin structure as shown in Fig. 5.

**Table 1:** Data of X-ray powder diffraction of H_5_Mo_11_VO_40_·8H_2_O

2*θ*	*I*/*I*_0_	*d* a (Å)
9.25	1.00	9.55
18.33	0.32	4.83
20.67	0.81	4.29
22.49	0.32	3.95
25.93	0.18	3.43
27.63	0.97	3.22
29.10	0.34	3.06
30.43	0.25	2.93
31.90	0.26	2.80
34.58	0.19	2.59
36.93	0.09	2.43
38.17	0.28	2.35
39.27	0.11	2.29

a *d* represents the lattice plane spacing calculated according to Bragg equation: 2*d* sin *θ* = *nλ*. (*λ* = 1.541 Å for Cu-Kα).

**Fig. 6:**
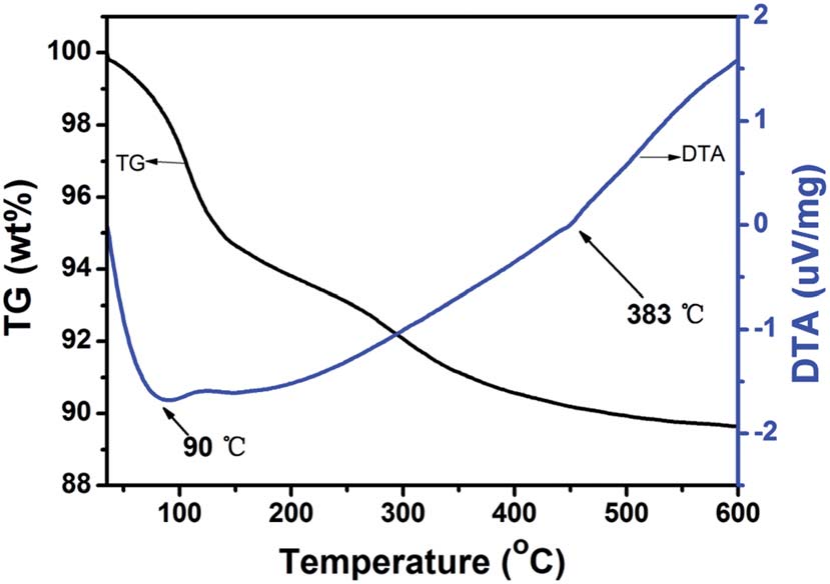
The TG-DTA curves of H_5_SiMo_11_VO_40_·8H_2_O.

The hydration degree and thermal stability of H_5_SiMo_11_VO_40_·8H_2_O was investigated by thermogravimetric (TG) and differential thermal analysis (DTA). Generally, heteropoly acids contain three kinds of crystallographic water: crystal water, protonized water and structure water. As illustrated in Fig. 6, The TG curve shows the total percent of the weight loss below 383 °C is 9.30%, which indicates that 10.5 molecules of water calculated are lost. Firstly, the loss of 4.9 molecules crystal water happen with corresponding to the weight loss of 4.61%, then, 4.1 molecules of protonized water are lost, and 1.4 molecules of crystal water are lost at last. Therefore, the accurate molecule formula of this heteropoly acid is (H_5_O_2_^+^)_2_H_3_[SiMo_11_VO_40_]·5H_2_O. What's more, it is noticed that there are two obvious peaks in DTA curve. The endothermic peak observed at 90 °C is believed to be the processes of the dehydration, and exothermic peak of 383 °C is attributed to the irreversible decomposition of heteropoly acid to individual oxides (SiO_2_, MoO_3_ and V_2_O_5_). It demonstrates that the decomposition temperature of H_5_SiMo_11_VO_40_·8H_2_O is 383 °C. According to the reported literature,^22,23^ the decomposition temperature of H_4_SiMo_12_O_40_·2H_2_O is 365 °C, indicating vanadium-containing heteropolyanion has better thermal stability than its parent acid.

**Fig. 7:**
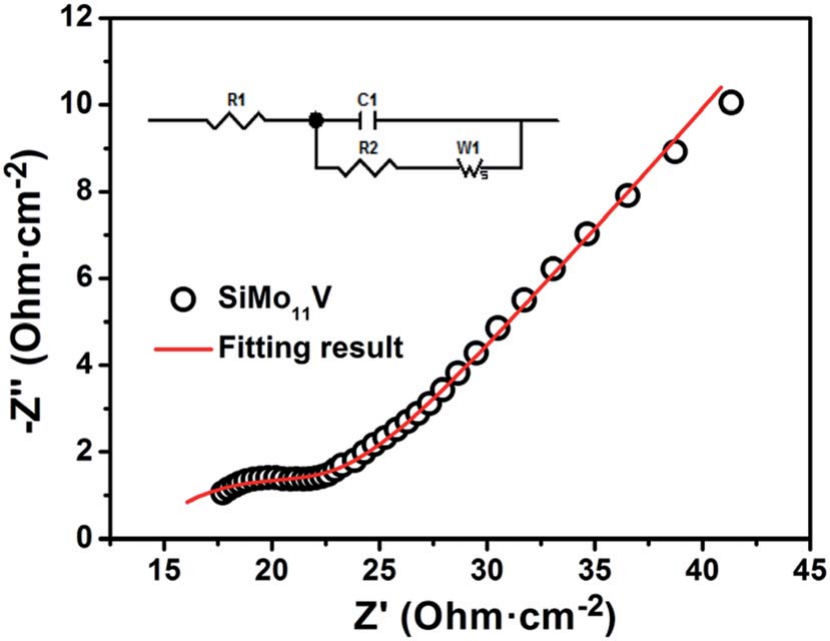
The electrochemical impedance spectrum of H_5_SiMo_11_VO_40_·8H_2_O at 26 °C and 70% relative humidity.

The proton conductivity is one of the most important properties of heteropoly acids. Fig. 7 is the electrochemical impedance spectrum of H_5_SiMo_11_VO_40_·8H_2_O, and inset is the equivalent circuit, where *R*_1_ is the bulk resistance, *C*_1_ represents a constant phase element of the double layers, *R*_2_ denotes the charge transfer resistance and *W*_1_ is the finite length Warburg element of solid diffusion. The proton conductivity of HPA is calculated using the following equation: *σ* = *L*/(*RS*) (*R* is the resistance, *L* is the thickness, and *S* is the area of the tablet). By calculation, the proton conductivity of H_5_SiMo_11_VO_40_·8H_2_O is 5.70 × 10^−3^ S cm^−1^ at 26 °C and 70% relative humidity. It shows a higher conductivity than H_4_SiMo_12_O_40_·12H_2_O (2.03 × 10^−4^ S cm^−1^),^24^ which illustrated that the incorporation of V element can increase hugely the proton conductivity of silicomolybdic acid. So, this heteropoly compound is a high proton conductor. Additionally, CPE for double layers from the equivalent circuit is 3.59 × 10^−3^ F, indicating that this acid has the potential for application in energy storage device, such as supercapacitors.

**Fig. 8:**
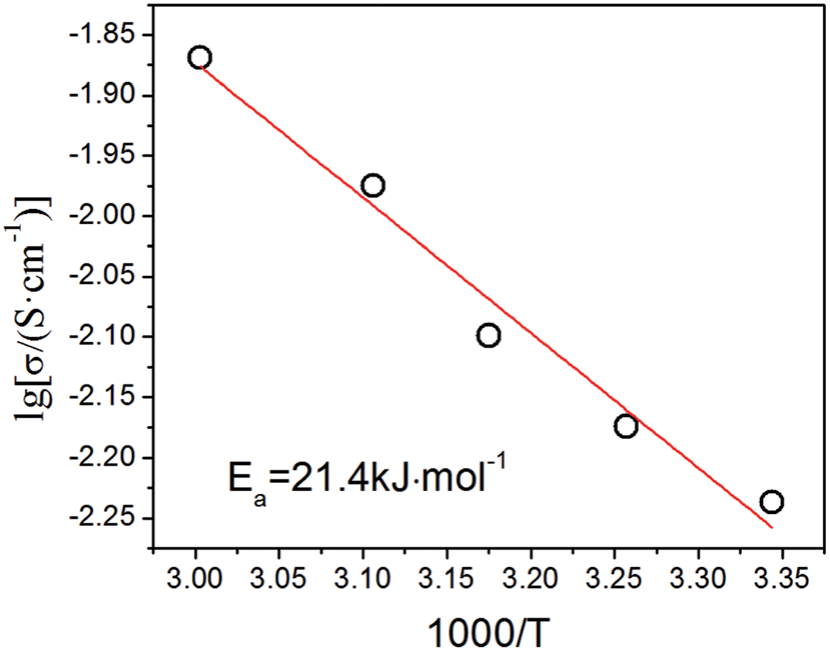
The Arrhenius plot for conductivity of H_5_SiMo_11_VO_40_·8H_2_O.

To investigate the relationship between proton conductivity and temperature, we measured the conductivity of this heteropoly acid at the temperature range of 26–60 °C. It is found that the conductivity value of this heteropoly acid enhances with higher temperature because the mobility of conducting species accelerates with the increase of temperature, which increases to 1.35 × 10^−2^ S cm^−1^ at 60 °C and 70% relative humidity. The relationship between proton conductivity and temperature is consistent with Arrhenius equation: *σ* = *σ*__0__ exp^(*E*_a_/*κT*)^. In this formula, *E*_a_ is the activation energy of proton conductivity, *σ*__0__ is the pre-exponential factor and *κ* presents the Boltzmann constant. As shown in Fig. 8, the activation energy of proton conductivity calculated from the slope of Arrhenius curve is 21.4 kJ mol^−1^.

So far, there are two major recognized proton conduction mechanisms: Vehicle and Grotthuss mechanism.^25,26^ In Vehicle mechanism, proton interacts with water molecules, which transfers in the form of hydrated hydrogen ions, such as H_3_O^+^, H_5_O_2_^+^ and H_9_O_4_^+^ species, similar to molecular diffusion. It differs from Grotthuss mechanism, in which a large amount of water can assist proton hopping from one proton carrier to a neighboring one down a chain of hydrogen-bonded network. Therefore, water plays a fairly important role in the process of proton mobility, and proton conductivity and Arrhenius parameters are strongly dependent on the water content.^27,28^ Generally, we distinguish them by the numerical value of activation energy and the type of hydrated hydrogen ions. The activation energy of Grotthuss mechanism is often less than 15 kJ mol^−1^, which is lower than that of Vehicle mechanism, whose is normally more than 20 kJ mol^−1^.^29,30^ The activation energy of this acid is 21.4 kJ mol^−1^. The acid protons and water molecules form H_5_O_2_^+^ that bridges the Keggin units owing to the low hydration levels (less than 10 water molecules of hydration per Keggin unit).^31^ Hence, we can speculate that the proton migration of H_5_SiMo_11_VO_40_·8H_2_O occurs by a mixing mechanism and Vehicle mechanism is predominant.

## 4. Conclusions

In this work, we have reported the synthesis of a vanadium-substituted heteropoly acid H_5_SiMo_11_VO_40_·8H_2_O, which was characterized by IR, XRD of powder, TG-DTA. This acid shows a good proton conductivity of 5.70 × 10^−3^ S cm^−1^ at 26 °C and 70% relative humidity. The proton conductivity enhances with the increase of temperature, and the proton conduction mechanism is dominated by Vehicle mechanism due to the activation energy of 21.4 kJ mol^−1^. It is a novel solid high proton conductor, which may be applied as solid electrolyte in the fields of fuel cell and supercapacitors.

## Conflicts of interest

There are no conflicts to declare.

## Supplementary Material
